# *QuickStats:* Percentage of Adults Aged 18–24 Years Who Currently Smoke Cigarettes* or Who Currently Use Electronic Cigarettes,^†^ by Year — National Health Interview Survey, United States, 2014–2018^§^

**DOI:** 10.15585/mmwr.mm6839a6

**Published:** 2019-10-04

**Figure:**
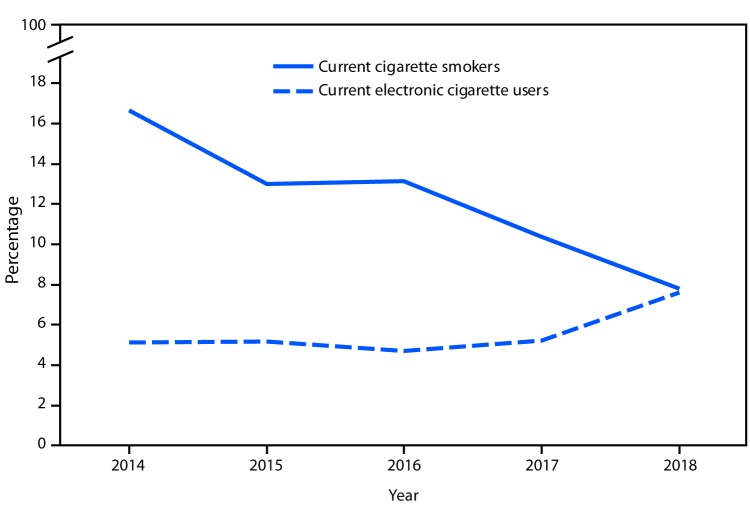
From 2014 to 2018, the percentage of adults aged 18–24 years who currently smoked cigarettes decreased from 16.7% to 7.8%. The percentage of adults in this age group who currently used electronic cigarettes increased from 5.1% to 7.6%.

**Source:** National Health Interview Survey, 2014–2018 data. https://www.cdc.gov/nchs/nhis.htm.

